# Spliceosome Mutations in Uveal Melanoma

**DOI:** 10.3390/ijms21249546

**Published:** 2020-12-15

**Authors:** Josephine Q.N. Nguyen, Wojtek Drabarek, Serdar Yavuzyigitoglu, Eva Medico Salsench, Robert M. Verdijk, Nicole C. Naus, Annelies de Klein, Emine Kiliç, Erwin Brosens

**Affiliations:** 1 Department of Ophthalmology, Erasmus MC University Medical Center Rotterdam, 3000 CA Rotterdam, The Netherlands; j.nguyen@erasmusmc.nl (J.Q.N.N.); w.drabarek@erasmusmc.nl (W.D.); s.yavuzyigitoglu.1@erasmusmc.nl (S.Y.); n.naus@erasmusmc.nl (N.C.N.); e.kilic@erasmusmc.nl (E.K.); 2 Department of Clinical Genetics, Erasmus MC University Medical Center Rotterdam, 3000 CA Rotterdam, The Netherlands; e.medicosalsench@erasmusmc.nl (E.M.S.); a.deklein@erasmusmc.nl (A.d.K.); 3 Department of Pathology, Section Ophthalmic Pathology, Erasmus MC Cancer Institute, Erasmus MC University Medical Center Rotterdam, 3000 CA Rotterdam, The Netherlands; r.verdijk@erasmusmc.nl; 4 The Rotterdam Eye Hospital, 3011 BH Rotterdam, The Netherlands; 5 Department of Pathology, Leiden University Medical Center, 2333 ZA Leiden, The Netherlands

**Keywords:** genetic, DNA repair, epigenetic, mutational analysis, chromosomes, prognosis, metastatic disease, therapy

## Abstract

Uveal melanoma (UM) is the most common primary intraocular malignancy of the eye. It has a high metastatic potential and mainly spreads to the liver. Genetics play a vital role in tumor classification and prognostication of UM metastatic disease. One of the driver genes mutated in metastasized UM is subunit 1 of splicing factor 3b (*SF3B1*), a component of the spliceosome complex. Recurrent mutations in components of the spliceosome complex are observed in UM and other malignancies, suggesting an important role in tumorigenesis. *SF3B1* is the most common mutated spliceosome gene and in UM it is associated with late-onset metastasis. This review summarizes the genetic and epigenetic insights of spliceosome mutations in UM. They form a distinct subgroup of UM and have similarities with other spliceosome mutated malignancies.

## 1. Introduction

Uveal melanoma (UM) is the most common primary ocular malignancy in adults [1]. The European incidence varies between approximately 2 to 8 per million with the southern parts of Europe having the lowest incidence and the northern parts having the highest [1]. It arises from uveal melanocytes and it is located in either the choroid (90.3%), ciliary body (6.1%) or iris (3.6%) [2]. UM usually presents with symptoms such as blurred vision, photopsia, floaters and visual field loss [3]. Other patients are asymptomatic and their UM are detected during routine ophthalmic examination. Diagnosis is made by an ophthalmologist using fundoscopy with additional techniques such as ultrasonography (US) and fluorescein angiography (FAG). UM diagnosis is confirmed to be histopathological if tissue is available from biopsy or enucleation. Metastatic disease is very rare at presentation [4] and in our cohort only 10 out of 808 (1.2%) UM patients presented with detectable metastatic disease at the time of diagnosis with ultrasonographic screening of the abdomen. This is most likely an underestimation, since not all metastasis can be detected with ultrasonography of the abdomen. UM can be a highly aggressive disease, with up to 25% of the UM patients developing metastases within 10 years after diagnosis [2]. Metastasis usually spreads via the hematogenous route, with the liver as the most common site [5]. However, patients also develop metastases after enucleation, suggesting the presence of micro metastases at an early stage during tumorigenesis [6]. Current treatments aim to conserve the eye and preserve vision, which varies from stereotactic radiotherapy, proton therapy and brachytherapy to enucleation [7].

Prognostication of the tumor can be staged using The American Joint Committee on Cancer (AJCC) Cancer Staging Manual, which uses the tumor-node-metastasis (TNM) classification [8]. This includes tumor size, basal diameter, tumor thickness, ciliary body involvement and extraocular tumor extension (EOE). Large tumors, presence of EOE and ciliary body involvement are associated with poor prognosis [2,9]. Histopathological parameters include tumor cell type, mitotic activity and closed extravascular matrix patterns. Epithelioid cell type, high number of mitoses and closed vascular loops are associated with poor survival rates [10,11].

**Figure 1:**
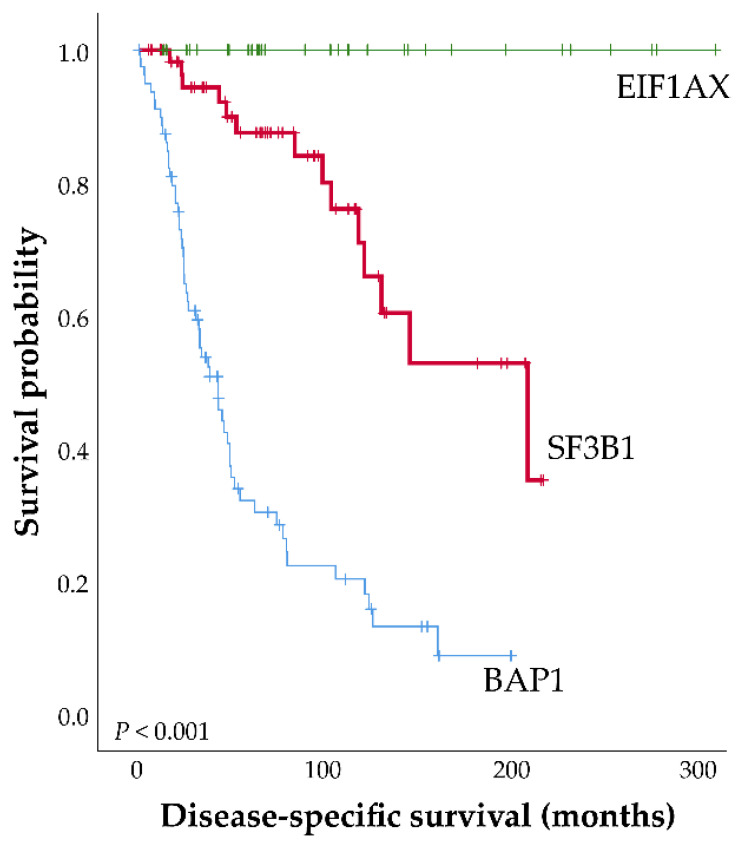
Disease-specific survival plot of Uveal melanoma (UM) patients, defined as the first date of treatment until the date of metastasis or until last follow-up. Patients were censored when they were lost to follow-up or when death from a cause other than UM occurred. Updated Kaplan-Meier curve of *SF3B1*-mutated UM adapted and updated from Yavuzyigitoglu et al. [21]. Three groups of patients with secondary driver gene mutations: *n* = 185; *EIF1AX*, *n* = 33; *SF3B1*, *n* = 63; *BAP1*, *n* = 89).

Gene mutations play an important role in prognosis. Activating mutations in *GNAQ* or *GNA11* are seen as primary driver oncogenes in UM and mutations in these genes are usually mutually exclusive [12]. *GNAQ* and *GNA11* encode for guanine nucleotide-binding protein alpha Q and guanine nucleotide-binding protein alpha-11 respectively, which are alpha subunits of the heterotrimeric G protein. These proteins transduce signals from G protein-coupled receptors (GPCR) to cellular signaling pathways [13]. Mutations in these genes occur in nearly all UM but are not associated with prognosis, because they are found not only in UM but also in benign blue nevi [14,15,16]. Recurrent mutations in *CYSLTR2* (Cysteinyl Leukotriene Receptor 2) and *PLCB4* (Phospholipase C Beta 4) were found in some of the cases lacking mutations in *GNAQ* or *GNA11* [17,18]. Secondary driver genes are strongly associated with prognosis and include *BAP1* [19], *EIF1AX* [20] and *SF3B1* [21]. *BAP1* is located on chromosome 3 (3p21) encoding the BRCA associated protein-1. BAP1 has many functions including protein de-ubiquitination, DNA damage repair, chromatin regulation and cell cycle regulation [22]. Loss of function mutations in *BAP1* are often concurrent with monosomy 3, often resulting in loss of BAP1 protein expression and are associated with poor prognosis [19,23]. In our cohort of 808 patients, *BAP1* mutations are seen in up to 82% of all metastasized UM patients and are correlated with monosomy 3 (87%). *EIF1AX* encodes the eukaryotic translation initiation factor 1A, an X-chromosomal protein and mutations in the N terminal region of this gene are found in approximately 20% of UM patients [20]. EIF1AX is a component of the 43S pre-initiation complex, which mediates the recruitment of the small 40S ribosomal subunit to the 5′ cap of mRNA to initiate translation of proteins [24]. The *EIF1AX* mutations are change-of-function mutations and they are associated with a more favorable prognosis in UM [20]. *SF3B1* encodes splicing factor 3B subunit 1 and mutations in the *SF3B1* gene occur in approximately 20% of UM cases [20,25,26]. Although mutations in *SF3B1* were initially supposedly associated with a better prognosis compared to *SF3B1* wild-type patients [25], our group has been able to demonstrate a biphasic development in *SF3B1* mutated UM [21]. A favorable prognosis was observed in the first few years after diagnosis. However, with longer follow-up, a decline in survival was observed due to late-onset metastasis (Figure 1).

## 2. SF3B1 and Other Spliceosome Mutations in Uveal Melanoma

This review focuses on elucidating the pathogenic features of this distinct subgroup of spliceosome gene mutated UM. RNA splicing is a fundamental process in eukaryotic species and accurate RNA splicing is essential for cell survival. Recurrent mutations in components of the spliceosome suggests these mutations are expressed and that a change in splicing accuracy that is caused by these mutations plays an important role in cancer [27].

**Figure 2:**
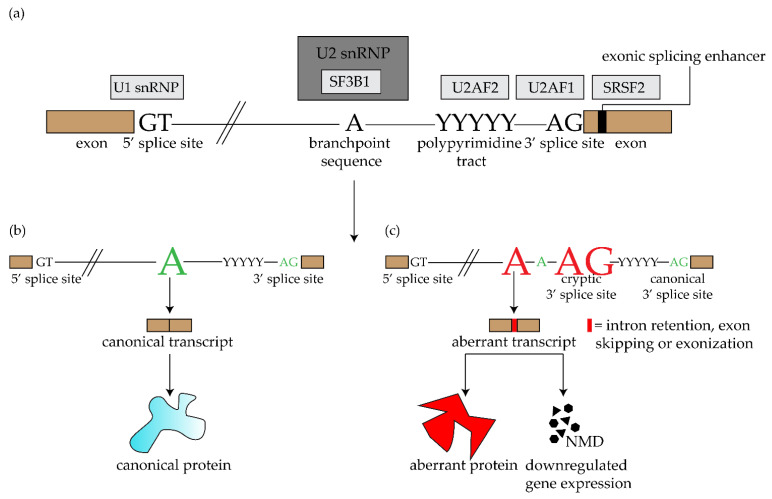
Splicing of pre-mRNA into mature mRNA. (**a**) Overview of different complexes in the spliceosome. (**b**) Splicing in wild-type SF3B1 (SF3B1^WT^) cells: SF3B1^WT^ binds to the branchpoint of the pre-mRNA, usually an adenosine. When this process is executed correctly, mature mRNA is formed which results in a canonical protein. (**c**) Splicing in mutated SF3B1 (SF3B1^MUT^) cells: SF3B1^MUT^ recognizes an alternative branchpoint, which leads to mis-splicing of the mRNA (e.g., intron retention, exon skipping and intronic extension of exon). The mis-spliced mRNA can be translated into aberrant proteins or be degraded by nonsense-mediated decay (NMD), resulting in downregulation of gene expression.

Mature mRNA is produced by removing the noncoding intronic sequences and ligating the coding sequences of pre-mRNA. This process—splicing—is regulated by the spliceosome. There are two types of this RNA/protein structure: the major spliceosome complex—involved in “general” or canonical splicing—and the minor spliceosome complex involved in non-canonical splicing (often resulting in tissue specific transcripts) [28,29]. Each of these spliceosome complexes include five crucial small nuclear ribonucleoprotein particles (snRNP): U1, U2, U4, U5 and U6 in the major spliceosome and U11, U12, U4atac, U5 and U6atac in the minor spliceosome. These particles recognize specific intronic consensus sequences: U2-type introns are recognized by the major spliceosome complex and U12 type sequences by the minor spliceosome. Canonical splicing occurs when consensus sequences in the intron: the 5′ donor splice site (5′ss), the 3′ acceptor splice site (3′ss), and the branchpoint sequence (BPS), are recognized by U1 snRNP, U2AF (U2 small nuclear RNA auxiliary factor) and U2 snRNP protein, respectively. U2AF is a heterodimer and together with the U2AF2 (U2 small nuclear RNA auciliary factor 2), it recognizes the AG dinucleotide sequence and the polypyrimidine (Py) tract of the 3′ss and coordinates the binding of the U2 snRNP at the BPS. Interaction of U1 and U2 snRNP generates junction of the 5′ end of the intron to the BPS (Figure 2). The 5′ss and 3′ss ligate together, which results in a mature mRNA product [30]. The U2 snRNP includes the SF3b1 subunit encoded by the *SF3B1* gene.

The *SF3B1* gene, hence an essential protein in the U2 snRNP complex, is the most frequently mutated spliceosome gene and mutations are present in various malignant diseases such as UM [26], myelodysplastic syndromes (MDS) [31], chronic lymphocytic leukemia (CLL) [32], breast cancer [33], pancreatic cancer [34] and mucosal melanoma (especially in anorectal and vulvovaginal melanomas) [35]. *SF3B1* mutations have an incidence of approximately 20% in UM [20,25,26], 42% in mucosal melanoma (60% in anorectal melanoma and 44% in vulvovaginal melanoma) [36], 7% in MDS (80% in refractory anemia with ring sideroblasts (RARS)-MDS, a subset of MDS) [31], 10% in CLL [32] and approximately 1.8% in breast cancer [37]. Mutated SF3B1 leads to deregulation of the U2 snRNP splicing due to an alternative usage of the 3′ss AG [38]. Mutated SF3B1 recognizes an alternative BPS located upstream of original BPS and this alternative BPS has a higher affinity to U2/SF3B1^mut^ snRNA compared to the canonical BPS. This alternate BPS usage results in aberrant splicing. The resulting aberrant spliced transcripts could be broken down due to nonsense-mediated decay (NMD) and result in downregulated gene expression or result in aberrant proteins with changed function (Figure 2) [39,40].

**Figure 3:**
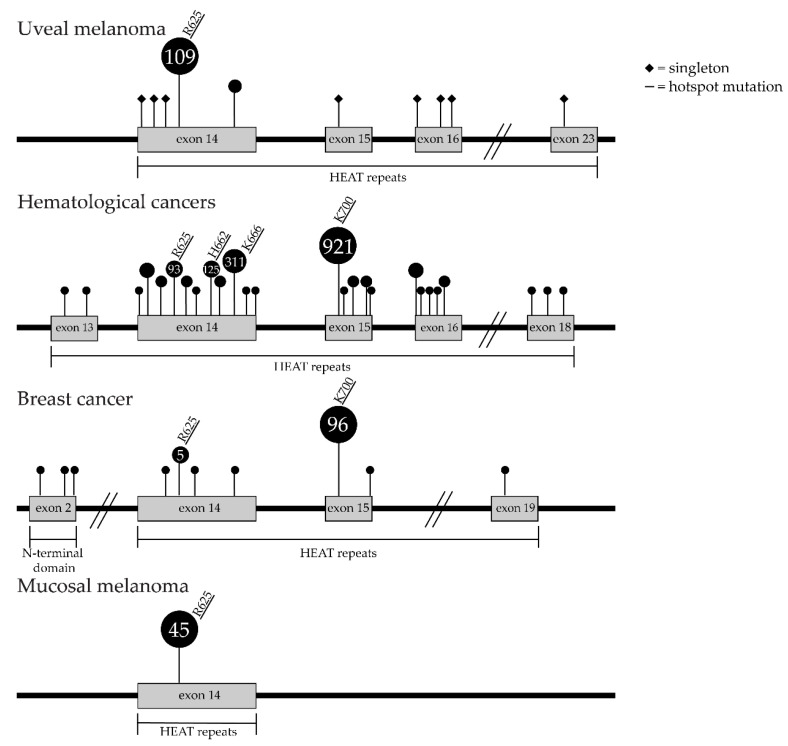
Overview of SF3B1 mutations in UM, hematological cancers, breast cancer and mucosal melanoma. Squares represent singleton mutations. Dots represent mutations seen at least two times. Underline represents hotspot mutations. Singleton mutations are only displayed for the UM cohort. For the hematological cancers and breast cancer cohorts only mutations with frequency of ≥5% are reported (see supplementary Table S1 for other data (Catalogue of Somatic Mutations in cancer v92, 27-08-2020, [46]).

Mutations in *SF3B1* are almost exclusively clustered in a specific repeat sequence motif: the 22 non-identical HEAT (Huntington, Elongation factor 3, protein phosphatase 2A, Targets of rapamycin 1) repeats domain region. Mutations in this motif have been shown to affect the interaction with the DDX46 (DEAD-Box Helicase 46) protein (*prp5* in yeast) necessary for stable association of U2 snRNP to the pre-mRNA [41]. In UM, hotspot mutations are mostly in the fifth HEAT repeat at codon position arginine (R) 625, although occasionally other mutations have also been reported (Table 1). In contrast, hotspot mutations in the sixth and seventh HEAT repeat codon position lysine (K) 666 and K700, respectively, are seen in the other cancers such as hematologic cancer and breast cancer (Figure 3) [31,33,42]. The few mutations outside the HEAT repeat domains do not display aberrant splicing, suggesting they have different effects on cancer cells or have no effect and are simply passenger mutations [39]. The outcome of a large study of 533 patients with MDS indicated that patients with *SF3B1* mutations were associated with a better overall survival and a lower risk of progression to acute myeloid leukemia compared to patients without *SF3B1* mutations [43]. Moreover, *SF3B1* mutations in MDS is an independent predictor of favorable outcome, although other studies indicated no significant effect on overall survival and were associated with shorter leukemia-free survival [44]. In breast cancer, *SF3B1* mutations are associated with poor prognosis in PR-negative and luminal B subgroups [45].

**Figure 4:**
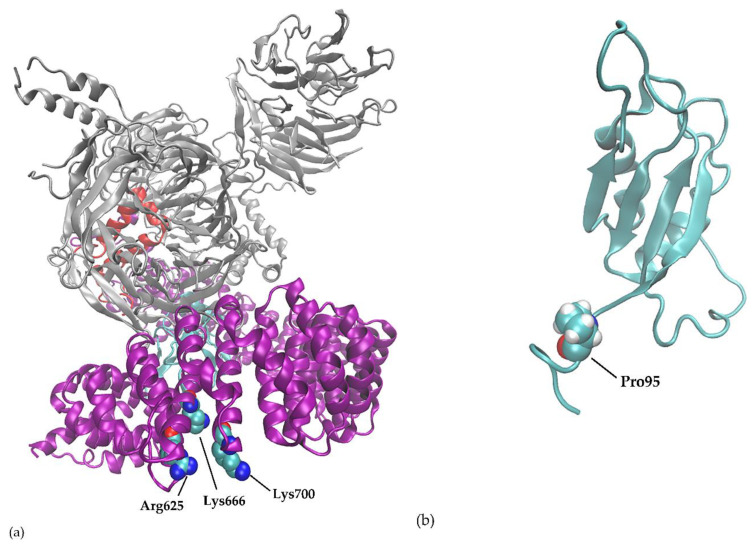
Three-dimensional view of the proteins. (**a**) SF3B1 protein with hotspot residue R625, K666 and K700. (**b**) SRSF2 protein with hotspot P95.

A variant of the 625R residue of SF3B1 is the most common spliceosome mutation seen in UM, but other mutations in another component of the spliceosome have also been reported [47]. Molecular modeling shows location of hotspot mutations in secondary structures of splicing genes (Figure 4).

A second, less frequently mutated spliceosome gene is in UM is the *SRSF2* (serine- and arginine-rich splicing factor 2). SRFSF2 is part of the SR protein family, which promotes spliceosome assembly by binding to exonic splicing enhancer (ESE) sequences. Van Poppelen et al. assessed *SRSF2* mutations in UM patients and found five patients with *SRSF2* mutations [48]. They all had in-frame deletions, but at 3 different locations. Two patients harbored in-frame deletions at protein residues 92–99, two at protein residues 92–100 and one patient harbored in-frame deletions at protein residues 173–179 [27,48]. Mutations in *SRSF2* occur in approximately 12% of MDS patients [31], however not as in-frame deletions but the more common missense mutations at hotspot P95 [31]. As in case of the *SF3B1* mutations, these in-frame deletions are typical for UM and are not observed in other malignancies where nucleotide change are more prevalent.

## 3. Chromosomal Anomalies and Epigenetic Changes Unique for SF3B1 UM

**Figure 5:**
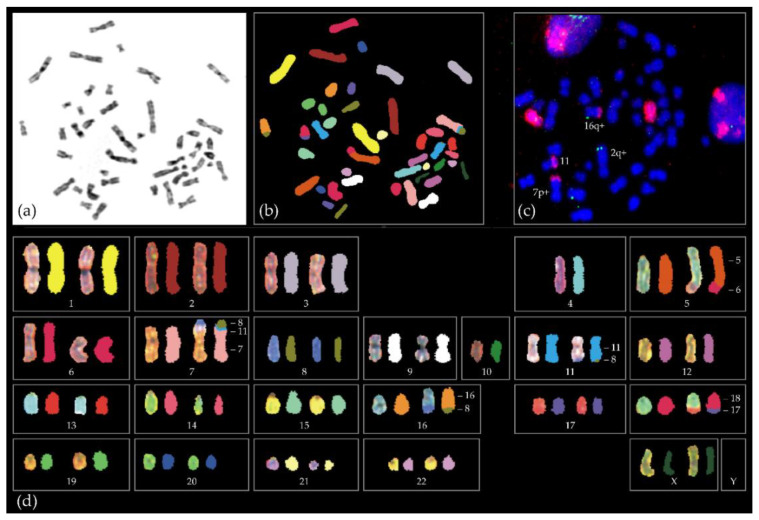
Example of the complex chromosome aberrations of a *SF3B1*-mutated tumour of a 38 year old female. Shown is (**a**) metaphase spread with DAPI staining, (**b**) spectral karyotyping in which each chromosome has a specific color, (**c**) red chromosome 8 paint and green telomeric 6q probe and (**d**) spectral karyotype showing exchanges between different chromosomes. For instance, part of chr6 (red) is attached to the bottom part (q-arm) of chromosome 5 (orange). The upper arm (p-arm) of chromosome 7 has material of chromosome 11 (blue) and 8 (green). Full karyotype in Naus et al. [53].

Chromosomal instability is characteristic for most cancers. In comparison to other cancers UM has relatively simple chromosomal anomalies [49]. In general, these chromosomal anomalies differ between secondary driver mutation types. *BAP1* mutated UM is strongly associated with loss of chromosome 3. This in contrast to UM without a *BAP1* anomaly, which retain both copies [47]. *BAP1* mutated UM are characterized by gains or losses of entire chromosomes or chromosome arms. *SF3B1* mutated UM karyotypes are more complex and characterized by multiple chromosomal structural variants (CSV) at the distal ends of chromosomes: loss of chromosomal region 6q (52%) and gain of chromosomal region 6p (85%) and 8q (73%) (Figure 5) [47,50]. Furthermore, *SF3B1* mutated UM are most likely to have more than 3 chromosomal structural variants (CSV) per tumor, with 70% of UM with >3 CSV harboring *SF3B1* mutations [50]. Between 20–36% of *SF3B1* mutated CLL tumors have deletions in chromosome 11q resulting in a worse prognosis [42,51]. For UM, we have updated the data used by Yavuzyigitoglu et al. [50], and found that 30 out of 63 (47.6%) of all *SF3B1* mutated UM with CSV analysis had aberrations in chromosome 11. Eight patients died due to UM, with a mean disease-specific survival of 46.5 months.

Aberrant methylation can cause aberrant gene regulation, which plays an important role in tumorigenesis. We and others have assessed aberrant methylation in metastatic uveal melanoma [47,52] and the results indicate a distinct hypomethylation pattern in *SF3B1* mutated patients compared to the *BAP1* mutated patients. Loss of expression of genes such as tumorsupressor genes or enhanced or ectopic expression of oncogenes could play a crucial role in spliceosome induced tumorigenesis, including UM.

## 4. Telomere Length in *SF3B1* Mutated UM

Aberrant transcripts in *SF3B1* mutations affect multiple cellular pathways, including the telomere maintenance pathway [54]. Telomeres are specific gDNA domains formed by repetitive DNA sequences (TTAGGG repeats) at the end of each chromosome and protect the ends of chromosomes from degradation and are essential for maintaining chromosomal stability [55]. Growth arrest and cellular senescence occur in cells when the TTAGGG repeats reach a critical length. Maintaining the telomerase length is regulated by enzyme telomerase (*TERT* gene) which is activated and upregulated in cancer cells for survival. Telomerase is detected in approximately 90% of all cancers [56]. Barthel et al. assessed telomere lengths in multiple cancer types including UM, and found shorter telomeres in tumors, including UM, compared to normal tissues [57]. *TERT* was expressed in 73% of all tumors and a third of these had *TERT* abnormalities, including *TERT* promotor mutations and amplification or chromosomal rearrangement. However, mutations in *TERT* promotor occur at extremely low frequency (1%) in UM compared to other types of ocular lesions [58]. Interestingly, the *TERT* promotor mutated *UM* had normal BAP1 staining and had wild type *SF3B1* as well as *EIF1AX*.

Our group has studied the length of telomeres in UM tumors harboring *SF3B1* and *BAP1* mutations. The total telomere length was significantly shorter in the *BAP1*^MUT^ group compared to the control group (*p* < 0.001). However, no significant difference in total telomere length was observed between the *SF3B1*^MUT^ group and control group (*p* = 0.054) or between the *SF3B1*^MUT^ and *BAP1*^MUT^ groups (Figure S1). We previously observed more chromosomal structural variants with recurrent distal breakpoints on chromosomes 6 and 8 in *SF3B1*^MUT^ UM, suggesting a different tumorigenesis in this group compared to *BAP1*^MUT^ UM [50]. Interestingly, we found that *SF3B1* mutated tumors had longer telomeres in chromosome 6 compared to controls and the CCCCAA repeats were significantly more expressed in chromosomes 6 p-arm and 8 p-arm in *SF3B1* mutated UM compared to *BAP1* mutated UM (*p* < 0.05). Previously, Doherty et al. [59] observed increased levels of DNA-PK (DNA-dependent protein kinase) in UM and this activity could indeed be instrumental for the observed increase in structural changes in *SF3B1*-mutated UM. However, in contrast to the M3G8q (monosomy 3, gain of 8q) subgroup of UM patients described in the Doherty paper where this gene is included in the amplified region, most of the 8q gains in tumors of *SF3B1* patients are gains of telomeric region of chromosome 8q and do not include the 8q11 region where DNA-PK is located. However, it would certainly be interesting to assess NHEJ (non-homologous end joining) expression upon having taken the mutational status in UM into account.

## 5. Therapeutic Opportunities

The prominent role of altered splicing in tumorigenesis makes it an alluring target for novel therapeutic approaches. Splicing inhibitors target specific components of the spliceosome which block spliceosome assembly [60]. Splicing inhibitors that interrupt U2 snRNP in joining the branch point sequence have been shown to target SF3B1. These include FR901464, pladienolide B and herboxidiene (Table 2) [60], Spliceostatin A, family of FR901464 and sudemycin. These splicing inhibitors have been evaluated in *SF3B1* mutant breast cancer cell lines and cells of MDS patients. Mutant *SF3B1* cells showed selective growth inhibition, altered splicing and increased cell death [37,61]. The effect of herboxidiene has been shown to modulate alternative pre-mRNA splicing in various human cancer cell lines [62]. H3B-8800, a pladienolide B derivative was developed and interacts directly with the SF3B1 complex [63]. It potently and preferentially kills spliceosome-mutant tumor cells. E7107, an analog of pladienolide B only affects wildtype SF3B1 and treating *SF3B1* mutated colorectal cancer cells with E7107 will result in cell death [64]. Our hypothesis is that the same results will also be seen in UM cells: E7107 exposure affecting wild type SF3B1, resulting in exclusively altered splicing by mutated SF3B1 and subsequent cell death. In vivo treatment of E7107 in *SRSF2* mutated murine myeloid leukemia cells resulted in preferential cell death of cells harboring mutant *SRSF2* [65,66,67]. This should be replicated in *SF3B1* mutated UM cell line to assess if the same results will be observed. E7107 has since been studied in two separate phase I clinical studies in patients with solid cancers [66,67]. A dose-dependent reversible inhibition of pre-mRNA was assessed in these patients. Unfortunately, unexpected toxicity of bilateral optic neuritis was reported in both studies with suspension of both trials [66,67].

Experiments with splicing inhibitors has resulted in potential target genes and therapies in splicing mutated malignancies. For example, Aird et al. has assessed the sensitivity of splicing inhibitors in cancer cell lines [72]. Cancer cells elude apoptosis through various pathways including the upregulation of anti-apoptotic *BCL2* (B-cell lymphoma 2) family genes. In this study, *BCL2A1* (BCL2 related protein A1) and *MCL1* (MCL1 apoptosis regulator), members of the *BCL2* family genes, were sensitive to E7107, whereas *BCL2L1* (BCL2 like 1), which encodes BCLxL (B-cell lymphoma-extra large), was highly resistant to E7107-induced splicing modulation. Furthermore, when E7107 was combined with BCLxL inhibitors, this enhanced the cytotoxicity in numerous cancer cell lines. These results indicate an increased cytotoxicity can be achieved with splicing inhibitors in combination with targeting *MCL1* and BCLxL. A second candidate gene is the MYC Proto-Oncogene, BHLH Transcription Factor (*MYC)*. This transcription factor is a principle regulator of the snRNP, ensuring proper splicing of mRNA [73]. Overexpression of MYC require cells to rely on upregulation of snRNP and PRMT5 (Protein Arginine Methyltransferase 5) to sustain splicing fidelity [73]. The MYC family consists of three genes: *c-MYC*, *l-MYC* and *n-MYC* [74]. One of these genes, the c-MYC gene, is located on chromosome 8q24 [75]. Intriguingly, *SF3B1* mutated UM are associated with gain of 8q, and proto-oncogene c-MYC is located in the 8q. Targeting *BUD31* (BUD31 homolog) in MYC driven tumors results in reduced cell viability by affecting the splicing machinery [76]. Third example is the Bromodomain Containing 9 (*BRD9*) gene. Mutated *SF3B1* recognizes aberrant deep intronic branchpoint in *BRD9*, a core component of the non-canonical BAF (ncBAF) chromatin remodeling complex [77]. This results into inclusion of an alternate exon with a premature termination codon and therefore depletion of BRD9. Loss of BRD9 promotes melanoma tumorigenesis through perturbation of ncBAF. When mis-splicing of *BRD9* in *SF3B1*-mutated cells was corrected, tumor growth was suppressed. Therefore, *BRD9* is a tumor suppressor in UM. Targeting mis-splicing of *BRD9* could therefore be a potential cancer therapy in *SF3B1* mutated malignancies.

CRISPR-Cas genome editing may prove a viable treatment option. The CRISPR-Cas uses guide RNAs to direct the Cas9 nuclease to the complementary target site where it cuts the double-stranded DNA, creating a break. The break will be repaired through homology recombination (HR) or non-homologous end joining (NHEJ), the latter results in the formation of indels [78]. *SF3B1* mutated UM does not lead to loss of splicing but aberrant splicing. Therefore, correcting the R625 hotspot with the CRISPR-Cas would result to normal splicing. The CRISPR-Cas technique has been studied in various cancer cell lines [79,80]. Moreover, results of a clinical trial using the CRISPR-Cas technique has recently been published [81]. However, delivery of the CRISPR gene therapy in vivo (off-target effects, targeted delivery, delivery efficiency and editing efficiency) remains challenging, and should be studied extensively before eventually applying this potential therapeutic method to patients with metastasized *SF3B1* mutated UM.

To conclude, spliceosome mutated UM has been shown to differ from other gene mutated UM in prognosis, pathogenesis, genetics and epigenetics. Furthermore, *SF3B1* mutated UM show similarities to other spliceosome mutated malignancies. This suggests that they should be considered as a different subgroup, most likely with different therapeutic options.

## Figures and Tables

**Table 1:** Table of *SF3B1* missense mutation frequency of uveal melanoma (UM) patients. Reference sequence: NM_012433.3(*SF3B1*); Build: hg38 (data from: Catalogue of Somatic Mutations in cancer v92, 27-08-2020, [46]).

Position	Exon	AA Mutation	CDS Mutation	Effect	Frequency (*n* = 122, %)
2: 197402759	14	p.R625H	c.1874G > A	Missense	61 (50%)
2: 197402760	14	p.R625C	c.1873C > T	Missense	38 (31%)
2: 197402759	14	p.R625L	c.1874G > T	Missense	7 (5.7%)
2: 197402760	14	p.R625G	c.1873C > G	Missense	1 (0.8%)
2: 197402759	14	p.R625P	c.1874G > C	Missense	1 (0.8%)
2: 197402760	14	p.R625S	c.1873C > A	Missense	1 (0.8%)
2: 197402775	14	p.M620V	c.1858A > G	Missense	1 (0.8%)
2: 197402767	14	p.E622D	c.1866G > C	Missense	1 (0.8%)
2: 197402766	14	p.Y623H	c.1867T > C	Missense	1 (0.8%)
2: 197402636	14	p.K666T	c.1997A > C	Missense	2 (1.6%)
2: 197402110	15	p.K700E	c.2098A > G	Missense	1 (0.8%)
2: 197401765	16	p.E783K	c.2347G > A	Missense	1 (0.8%)
2: 197401771	16	p.D781N	c.2341G > A	Missense	1 (0.8%)
2: 197401887	16	p.G742D	c.2225G > A	Missense	1 (0.8%)
2:197396227	23	p.C1123Y	c.3368G > A	Missense	1 (0.8%)

**Table 2:** Overview of different groups of splicing inhibitors.

Splicing Inhibitors	Features	Trials
FR901464 spliceostatins A-G, meamycins	Targets the U2 snRNP of SF3B1; isolated from fermentation broth of bacterium *Pseudomonas* sp. No. 2663 [68].	Preclinical trial in cancer cell lines [68]
Herboxidiene	Targets the U2 snRNP of SF3B1; isolated from *Streptomyces chromofuscus* ATCC 49,982 [69].	Preclinical trial in cancer cell lines [62]
Pladienolide B pladienolides A-G, H3B-8800, E7107	Targets the U2 snRNP of SF3B1; isolated from bacterium *Streptomyces platensis* [70].	Two phase I trials of E7107 on solid tumors, suspended due to side effects [66,67]
Sudemycin	Targets SF3B1; pharmacophore between FR901464 and pladienolide B [71].	Preclinical trial in cancer cell lines and tumor samples [71]

## Supplementary Materials

The following are available online at https://www.mdpi.com/1422-0067/21/24/9546/s1, Figure S1: Multiple comparison using TelomereHunter data, Table S1: Missense mutation frequency of *SF3B1* mutation in patients with hematological cancers, breast cancer and mucosal melanoma.

## Abbreviations

AJCC	American Joint Committee on Cancer
BAP1	BRCA1 associated protein 1
BCL2	B-cell lymphoma 2
BCL2A1	BCL2 related protein A1
BCL2L1	BCL2 like 1
BCLxL	B-cell lymphoma-extra large
BPS	Branchpoint sequence
BRD9	Bromodomain containing 9
BUD31	BUD31 homolog
CLL	Chronic Lymphocytic Leukemia
CSV	Chromosomal structural variants
CYSLTR2	Cysteinyl Leukotriene Receptor 2
DDX46	DEAD-Box Helicase 46
DNA-PK	DNA-dependent protein kinase
EIF1AX	Eukaryotic Translation Initiation Factor 1A X-Linked
EOE	Extraocular tumor extension
ESE	Exonic splicing enhancer
FAG	Fluorescein Angiography
GNAQ	G protein Subunit Alpha Q
GNA11	G protein Subunit Alpha 11
GPCR	G protein-coupled receptors
HEAT	Huntington, Elongation factor 3, protein phosphatase 2A, targets of rapamycin 1
HR	Homology recombination
K	Lysine
M3G8q	Monosomy 3, gain of 8q
MCL1	MCL1 apoptosis regulator
MDS	Myelodysplastic syndromes
MUT	Mutated
MYC	MYC proto-oncogene, BHLH transcription factor
ncBAF	Non-canonical BAF
NHEJ	Non-homologous end joining
NMD	Nonsense mediated decay
PLCB4	Phospholipase C Beta 4
PRMT5	Protein Arginine Methyltransferase 5
Py	Polypyrimidine
R	Arginine
RARS	Refractory anemia with ring sideroblasts
SF3B1	Splicing Factor 3b Subunit 1
SRSF2	Serine- and arginine-rich splicing factor 2
snRNP	Small nuclear ribonucleoprotein particles
TERT	Telomerase reverse transcriptase
TNM	Tumor-node-metastasis
U2AF	U2 small nuclear RNA auxiliary factor
U2AF2	U2 small nuclear RNA auxiliary factor 2
UM	Uveal melanoma
US	Ultrasonography
WT	Wild type
