# Supplementary material for: Spliceosome Mutations in Uveal Melanoma
Source: Int J Mol Sci. 2020 Dec 15;21(24):9546. doi: 10.3390/ijms21249546 (PMC7765440; doi:10.3390/ijms21249546)
Supplement: Supplementary file 1 [file ijms-21-09546-s001.zip › review UM spliceosome supplementary FINAL.docx]

**Table S1.** Table of missense mutation frequency of *SF3B1* mutation in patients with hematologic cancers, breast cancer and mucosal melanoma. Singleton mutations were excluded. Reference sequence: NM_012433.3(SF3B1); Build: hg38 (data from: Catalogue of Somatic Mutations in cancer v92, 27-08-2020, [1]).

| **Malignancy** | **Position** | **Exon** | **AA mutation** | **CDS mutation** | **Frequency** |
| --- | --- | --- | --- | --- | --- |
| Hematological cancer | 2:197403005 | 13 | p.D586H | c.1756G>C | 4 |
|  | 2:197402981 | 13 | p.E592K | c.1774G>A | 10 |
|  | 2:197402820 | 14 | p.G605S | c.1813G>A | 2 |
|  | 2:197402767 | 14 | p.E622D | c.1866G>T | 42 |
|  | 2.197402767 | 14 | p.E622D | c.1866G>C | 21 |
|  | 2:197402765 | 14 | p.Y623C | c.1868A>G | 12 |
|  | 2:197402757 | 14 | p.N626Y | c.1876A>T | 10 |
|  | 2:197402757 | 14 | p.N626D | c.1876A>G | 5 |
|  | 2:197402756 | 14 | p.N626I | c.1877A>T | 4 |
|  | 2:197402756 | 14 | p.N626S | c.1877A>G | 3 |
|  | 2:197402757 | 14 | p.N626H | c.1876A>C | 2 |
|  | 2:197402659 | 14 | p.W658C | c.1974G>C | 2 |
|  | 2:197402645 | 14 | p.T663I | c.1988C>T | 17 |
|  | 2:197402625 | 14 | p.Q670E | c.2008C>G | 2 |
|  | 2:197402619 | 14 | p.A672T | c.2014G>A | 2 |
|  | 2:197402107 | 15 | p.V701F | c.2101G>T | 7 |
|  | 2:197402098 | 15 | p.I704F | c.2110A>T | 10 |
|  | 2:197402097 | 15 | p.I704S | c.2111T>G | 4 |
|  | 2:197402097 | 15 | p.I704N | c.2111T>A | 4 |
|  | 2:197401989 | 15 | p.G740E | c.2219G>A | 17 |
|  | 2:197401990 | 15 | p.G740R | c.2218G>A | 2 |
|  | 2:197401985 | 15 | p.K741N | c.2223G>T | 3 |
|  | 2:197401987 | 15 | p.K741E | c.2221A>G | 2 |
|  | 2:197401887 | 16 | p.G742D | c.2225G>A | 70 |
|  | 2:197401882 | 16 | p.A744P | c.2230G>C | 2 |
|  | 2:197401860 | 16 | p.G751V | c.2252G>T | 2 |
|  | 2:197401788 | 16 | p.R775Q | c.2324G>A | 2 |
|  | 2:197401788 | 16 | p.R775L | c.2324G>T | 2 |
|  | 2:197401770 | 16 | p.D781G | c.2342A>G | 6 |
|  | 2:197400934 | 18 | p.L833F | c.2499A>T | 3 |
|  | 2:197400849 | 18 | p.E862K | c.2584G>A | 2 |
|  | 2:197400752 | 18 | p.D894G | c.2681A>G | 3 |
|  | 2:197400753 | 18 | p.D897N | c.2680G>A | 2 |
| Breast cancer | 2:197423948 | 2 | p.Q19E | c.55C>G | 2 |
|  | 2:197423813 | 2 | p.E64K | c.190G>A | 2 |
|  | 2:197423810 | 2 | p.D65N | c.193G>A | 2 |
|  | 2:197402766 | 14 | p.Y623H | c.1867T>C | 2 |
|  | 2:197402757 | 14 | p.N626D | c.1876A>G | 2 |
|  | 2:197402637 | 14 | p.K666Q | c.1996A>C | 2 |
|  | 2:197401985 | 15 | p.K741N | c.2223G>C | 2 |
|  | 2:197400349 | 19 | p.T935K | c.2804C>A | 2 |
| Mucosal melanoma | 2:197402759 | 14 | p.R625H | c.1874G>A | 27 |
|  | 2:197402760 | 14 | p.R625C | c.1873C>T | 9 |
|  | 2:197402759 | 14 | p.R625L | c.1874G>T | 7 |
|  | 2:197402760 | 14 | p.R625S | c.1873C>A | 2 |


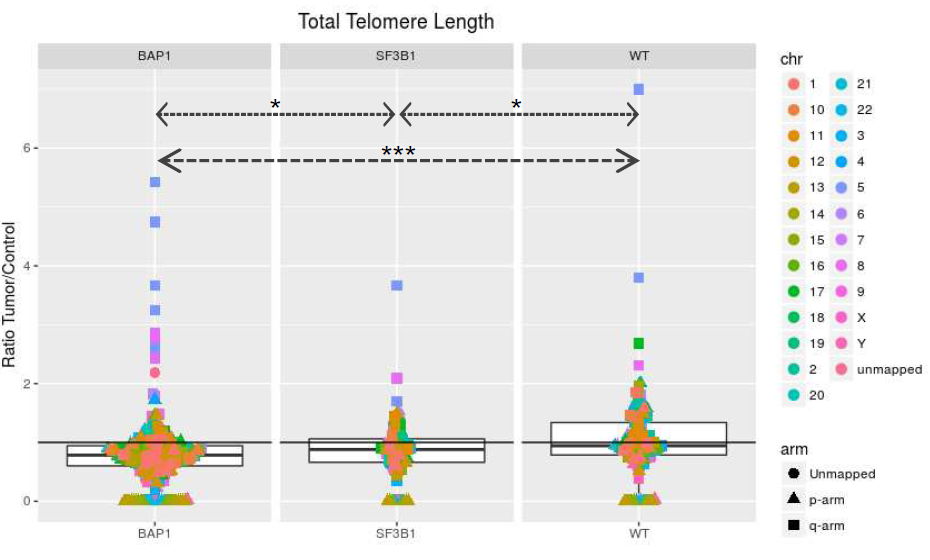


**Figure S1.** Multiple comparison using TelomereHunter data.

WGS data of 12 UM samples from COSMIC cohort published by Furney et al. [2] was used to analyze the telomere length using TelomereHunter software and post hoc test were done with the Tukey test [3]. Two samples harbored a SF3B1 mutation. One sample was excluded due to a double mutational status BAP1 and SF3B1. Samples were grouped based on mutational status on the x-axis (WT = only GNAQ or GNA11 mutation). The TL ratio (TL tumor/control) was plotted on the y-axis. Each chromosome is represented with a distinct color along with the different chromosomal arm: circle for q-arm and triangle for p-arm. The three asterisks represent significant statistical differences (p < 0.001) between groups and one asterisk represents no statistical differences (p > 0.05).

1. Tate, J.G.; Bamford, S.; Jubb, H.C.; Sondka, Z.; Beare, D.M.; Bindal, N.; Boutselakis, H.; Cole, C.G.; Creatore, C.; Dawson, E., et al. COSMIC: the Catalogue Of Somatic Mutations In Cancer. *Nucleic Acids Res* **2019**, *47*, D941-D947.

2. Furney, S.J.; Pedersen, M.; Gentien, D.; Dumont, A.G.; Rapinat, A.; Desjardins, L.; Turajlic, S.; Piperno-Neumann, S.; de la Grange, P.; Roman-Roman, S., et al. SF3B1 mutations are associated with alternative splicing in uveal melanoma. *Cancer Discov* **2013**, *3*, 1122-1129, doi:10.1158/2159-8290.CD-13-0330.

3. Feuerbach, L.; Sieverling, L.; Deeg, K.I.; Ginsbach, P.; Hutter, B.; Buchhalter, I.; Northcott, P.A.; Mughal, S.S.; Chudasama, P.; Glimm, H., et al. TelomereHunter - in silico estimation of telomere content and composition from cancer genomes. *BMC Bioinformatics* **2019**, *20*, 272, doi:10.1186/s12859-019-2851-0

10.1186/s12859-019-2851-0 [pii].
